# Simultaneous electrophysiological and morphological assessment of functional damage to neural networks *in vitro* after 30–300 g impacts

**DOI:** 10.1038/s41598-019-51541-x

**Published:** 2019-10-18

**Authors:** Edmond A. Rogers, Guenter W. Gross

**Affiliations:** 1 Department of Biological Sciences and Center for Network Neuroscience University of North Texas, Denton, TX 76203 USA; 2 Department of Basic Medical Sciences, School of Veterinary Medicine, Purdue University, West Lafayette, IN 47907 USA; 3 Weldon School of Biomedical Engineering, Purdue University, West Lafayette, IN 47907 USA

**Keywords:** Extracellular recording, Trauma, Experimental models of disease, Biomedical engineering, Biological physics

## Abstract

An enigma of mild traumatic brain injury are observations of substantial behavior and performance deficits in the absence of bleeding or other observable structural damage. Altered behavior and performance reflect changes in action potential (AP) patterns within neuronal networks, which could result from subtle subcellular responses that affect synaptic efficacy and AP production. The aim of this study was to investigate and quantify network activity changes after simulated concussions *in vitro* and therewith develop a platform for simultaneous and direct observations of morphological and electrophysiological changes in neural networks. We used spontaneously active networks grown on microelectrode arrays (MEAs) to allow long-term multisite monitoring with simultaneous optical observations before and after impacts delivered by a ballistic pendulum (30 to 300 g accelerations). The monitoring of AP waveshape templates for long periods before and after impact provided an internal control for cell death or loss of cell-electrode coupling in the observed set of neurons. Network activity patterns were linked in real-time to high power phase contrast microscopy. There was no overt loss of glial or neuronal adhesion, even at high-g impacts. All recording experiments showed repeatable spike production responses: a loss of activity with recovery to near reference in 1 hr, followed by a slow activity decay to a stable, level plateau approximately 30–40% below reference. The initial recovery occurred in two steps: a rapid return of activity to an average 24% below reference, forming a level plateau lasting from 5 to 20 min, followed by a climb to within 10% of reference where a second plateau was established for 1 to 2 hrs. Cross correlation profiles revealed changes in firing hierarchy as well as in Phase 1 in spontaneous network oscillations that were reduced by as much as 20% 6–8 min post impact with only a partial recovery at 30 min. We also observed that normally stable nuclei developed irregular rotational motion after impact in 27 out of 30 networks. The evolution of network activity deficits and recovery can be linked with microscopically observable changes in the very cells that are generating the activity. The repeatable electrophysiological impact response profiles and oscillation changes can provide a quantitative basis for systematic evaluations of pharmacological intervention strategies. Future expansion to include fluorescent microscopy should allow detailed investigations of damage mechanisms on the subcellular level.

## Introduction

The nervous system consists of tissue displaying highly dynamic electrophysiological properties, which underlie all behavior, performance, and perceptions. Investigations of chemical or physical insults to the nervous system require analyses of functional (i.e. electrophysiological) data for a thorough understanding of the type of damage experienced and for the design of realistic recovery strategies. TBI damage resulting from concussions is frequently microscopic and often causes no bleeding. Diffuse axonal damage has been demonstrated^1,2^; and includes inflammatory responses^3^ and calpain-mediated cytoskeletal changes^4^. Recent reviews^5,6^ highlight the need for cellular and sub cellular models to investigate changes in structure and function of both neurons and glia. It is evident that this complex pathology requires research on all levels: from studies of holistic brain injury to damage of cellular and even synaptic structures.

The simultaneous monitoring of functional and morphological/subcellular changes represent an essential component of TBI investigations. Although admirable attempts have been made to measure electrophysiological changes in animals after mechanical trauma^7,8^, direct before and after tissue damage evaluations are not possible, and must be inferred from sham-operated control studies. Also, monitoring changes in an electrophysiologically characterized network with optical access to the very cells that are generating the activity cannot be done *in vivo* and requires *in vitro* platforms.

In this paper, we present data from the simultaneous morphological and functional monitoring of neural tissue subjected to high accelerations. This was achieved *in vitro* with networks growing on transparent microelectrode arrays (MEAs) that allow multisite recording of action potential traffic as well as high resolution morphological and subcellular observations. Both vital modalities can be monitored for many hours, even days, before and after the experimental manipulation. We selected tangential acceleration provided by a ballistic pendulum (BPA) consisting of a striker arm and a target arm holding the MEA-recording chamber assembly. The target arm was allowed to swing freely to dissipate the transfer of momentum without introducing secondary influences. The arm was arrested by hand at its peak and returned gently to its original position. We consider this method a direct approach for creating pure acceleration conditions following an impact (impulse) without shock waves and tissue distortions other than those created by the acceleration.

In the domain of traumatic brain injury, the use of a ballistic pendulum is not new and was applied by Bakay *et al*.^9^ in 1977 for investigations of cerebral concussions. Lucas and Wolf^10^ used a prototype BPA to study cell death in spinal cord networks cultured in flasks. For tangential impacts, most neuronal death occurred within 15 min with a threshold of 450 g, and reached 50% at 1,100 g. The NMDA channel blocker ketamine at 100 uM substantially reduced neuronal cell death. Impacts perpendicular to the plane of growth did not cause cell death. However, the flask medium had to be removed for 30 sec during the impact and multichannel electrophysiological monitoring was not possible at that time. Our focus was to mimic mild traumatic brain injury by showing electrophysiological changes in network activity in the absence of medium removal and hydrodynamic stress. We used stainless-steel chambers with a medium volume of 5 ml that was not removed during the impact. It was hoped damage profiles could be established that would provide a quantitative basis for studies of pharmacological, chemical, and physical interventions to enhance recovery, or to minimize damage if applied before the rapid acceleration exposure.

We report that repeatable electrophysiological response profiles emerge after impact and that network oscillations decrease by 20% with only partial recovery in 30–40 min. We also have made the surprising observation that, in almost all experiments, the normally stable nucleus shows slow, irregular rotational movements after impact.

## Results

### Electrophysiological responses

**Figure 1:**
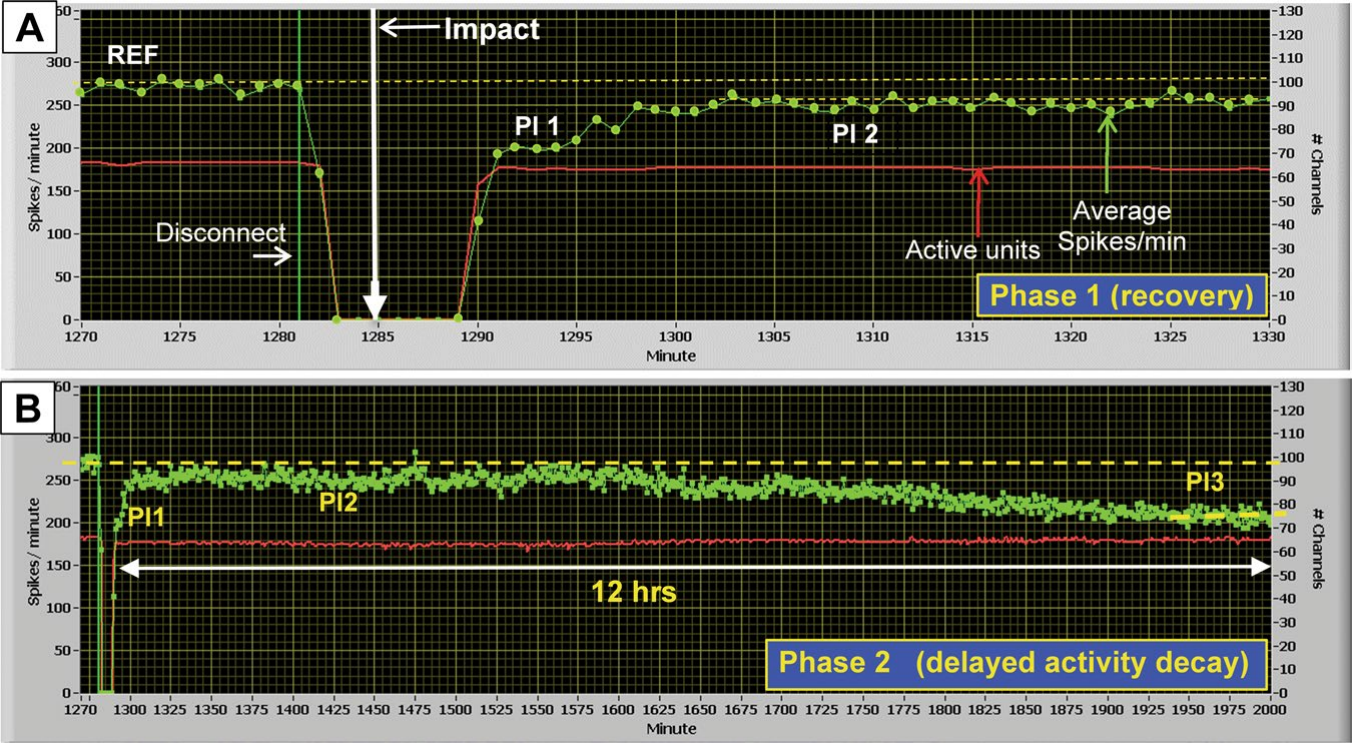
Raw data display of dominant response profile. Impact is denoted by vertical arrow. The red line indicates the number of wave-shape discriminated active units detected per min (criteria: minimum of 10 spikes/min). Green dots (left ordinate) represent consecutive one- minute averages of spike activity from these units. The activity gap reflects the chamber disconnect from the recording equipment. (**A**) Phase 1. A brief (5 min) partial activity recovery to 71% of reference (Pl 1), is followed by a 3 min climb to within 90% of reference, where activity remains stable for ~300 min (Pl 2). Dashed yellow lines highlight activity plateaus. REF is the native culture spontaneous activity. (**B**) Phase 2. Condensed time scale of Phase 1 showing the 300 min Plateau 2 stabilization followed by a subsequent decay to Plateau 3, 32% below reference. The asymmetry of impact time in the 7 min disconnect period (**A**) reflects chamber re-attachment to the microscope, medium circulation, and amplifiers as well as a two-minute temperature stabilization time. (example: ER52).

Electrophysiological responses showed common characteristics relative to the reference activity before impact. The recovery can be described in terms of two activity plateaus in the first two hours. The impact response is shown in Fig. 1, which plots average network spike production per minute and the total number of discriminated (via template matching, Plexon Inc.) active units as a function of time. The response profiles are reproducible and appear even when some units are lost from the recording set. It is important to emphasize that amplifiers, after impact and reassembly, were not switched on until the temperature of the chamber reached 37 °C. Plateau 1 is therefore not a temperature effect but represents a deficit in electrophysiological activity. Pl 2 does not reach the reference level but shows a variable activity deficit that is summarized in Table 1. Pl 2 is followed by a gradual, further activity decrease to a stable state (Pl 3). This decrease is not a normal decay of activity during long hours in the recording chamber. With the methods described, control experiments showed highly stable activity for much longer periods. Once Plateau 3 was reached, a recovery was not seen in a maximum observation period of 24 hours.

**Table 1:** Data Set and Summary of Responses.

EXP #								PHASE				PHASE 2	
								Plateau 1		Plateau 2		Plateau 3	
	age (div)	peak g	#of Imp	toal g	#of units	unit loss	% loss	% dec from ref	durat (min)	% dec from ref	durat (min)	time to Pl 3	% dec from ref
ER05	23	250	1	**250**	35	0	0	−14%	12	−8%	>50	nm	nm
ER09	22	50	1	**50**	49	0	0	−11%	4	−8%	10	nm	nm
ER10	23	250	5	**1250**	48	18	30	−34%	14	−20%	60	17 h	−50%
ER12	30	200	5	**1000**	100	0	0	−28%	8	−17%	>40	nm	nm
ER18	46	250	1	**250**	43	0	0	−15%	6	−5%	35	nm	nm
ER21	49	200	3	**600**	11	0	0	−17%	6	−5%	60	nm	nm
ER24	31	200	5	**1000**	45	0	0	−30%	5	−19%	30	6 h	−69%
ER28	32	100	5	**500**	29	0	0	−21%	4	−6%	450	9 h	−12%
ER30	88	100	5	**500**	15	4	27	−28%	5	−14%	100	10 h	−37%
ER39	22	100	5	**500**	20	1	5	−19%	5	−5%	10	16 h	−38%
ER50	26	250	5	**1250**	28	0	0	−29%	6	−9%	270	11 h	−15%
ER52	30	250	5	**1250**	65	1	2	−28%	5	−10%	300	17 h	−32%

div: days *in vitro* (age of network); peak g: per single impact; # of Im: number of impacts; total g: sum of g exposure at 5–8 sec intervals; # of un.: number of selected template- discriminated waveshapes (representing single neurons); unit loss: number of templates (neurons) lost from data set as logged approximately 10 min after impact. For pl 1, 2, and 3 please refer to Fig. 1; ref: reference activity before impact; time to Pl 3: time between impact and the formation of a level activity plateau 3; nm: not measured (termination of experiment or use for a second impact episode).

Table 1 summarizes profile features from 12 experiments subjected to their first impact episode. As described in methods, multiple impacts at 5–8 sec intervals were used to generate high g exposures, an experimental step designed to minimize MEA breakage. In all cases Plateau 1 showed activity decreases, which ranged from 11 to 34%. The significance of this Table is to convince the reader that the Phase 1 electrophysiological profiles are qualitatively reproducible despite differences in culture age, the number of units recorded per network, and the number of units lost after impact. The data set is arranged chronologically with missing numbers denoting morphological or control experiments as well as failure due to MEA breakage or life support problems. Whereas Phase 1 has received quantitative attention in subsequent figures and tables, Phase 2 is at this point less defined and requires more observation. It is included in the Table because the long-term trends are reproducible, possibly reflecting late onset damage.

**Figure 2:**
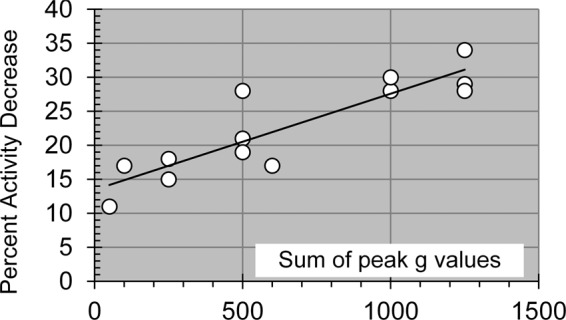
Plateau 1 activity deficits as a function of acceleration (g). Peak g values are shown on the x axis and represent the sum of accelerations obtained from multiple impacts, separated by 5–8 sec. R^2^ = 0.8. The function is not defined below 30 g. The plateau activity is represented as a percent decrease from reference (pre-impact) activity.

If the Plateau 1 decreases listed in Table 1 are plotted against the sum of the peak g’s during the exposure episode (5–8 seconds between impacts), it is evident that this electrophysiological deficit is a function of the total g exposure (Fig. 2). The graph includes single impacts as well as 3 and 5 impacts (see Table 1). Damage is clearly correlated with increasing g-exposure.

For a variety of reasons, experiments were not always performed at the same time after chamber assembly. In some experiments, networks were left idling overnight to provide long control segments and were then used on the following day. Figure 2 does not include the time after chamber assembly when experiments were performed and it is important to demonstrate that loitering time in the chamber does not affect the phase 1 responses. This is shown in Table 2 with three sets of two experiments exposed to identical g forces but at greatly different times after chamber assembly. In no case are the longer chamber times associated with enhanced plateau 1 activity reductions. Within the time frame of 2040 min, a trend to greater activity decreases with time in the flow chamber is not seen.

**Table 2:** Network time in recording chamber does not affect responses to impact.

Exp #	Number of Units	Impact [g]	# of impacts	Total g	Pl1% Activity Loss	Time in Chamber*(min)
ER18	44	250	1	250	−15%	60
ER22	25	250	1	250	−17%	1606
ER24	45	200	5	1000	−30%	80
ER12	100	200	5	1000	−28%	2040
ER10	48	250	5	1250	−34%	201
ER52	65	250	5	1250	−28%	1285

*Time of impact after chamber assembly.

### Impact episodes separated by several hours show enhanced damage

**Figure 3:**
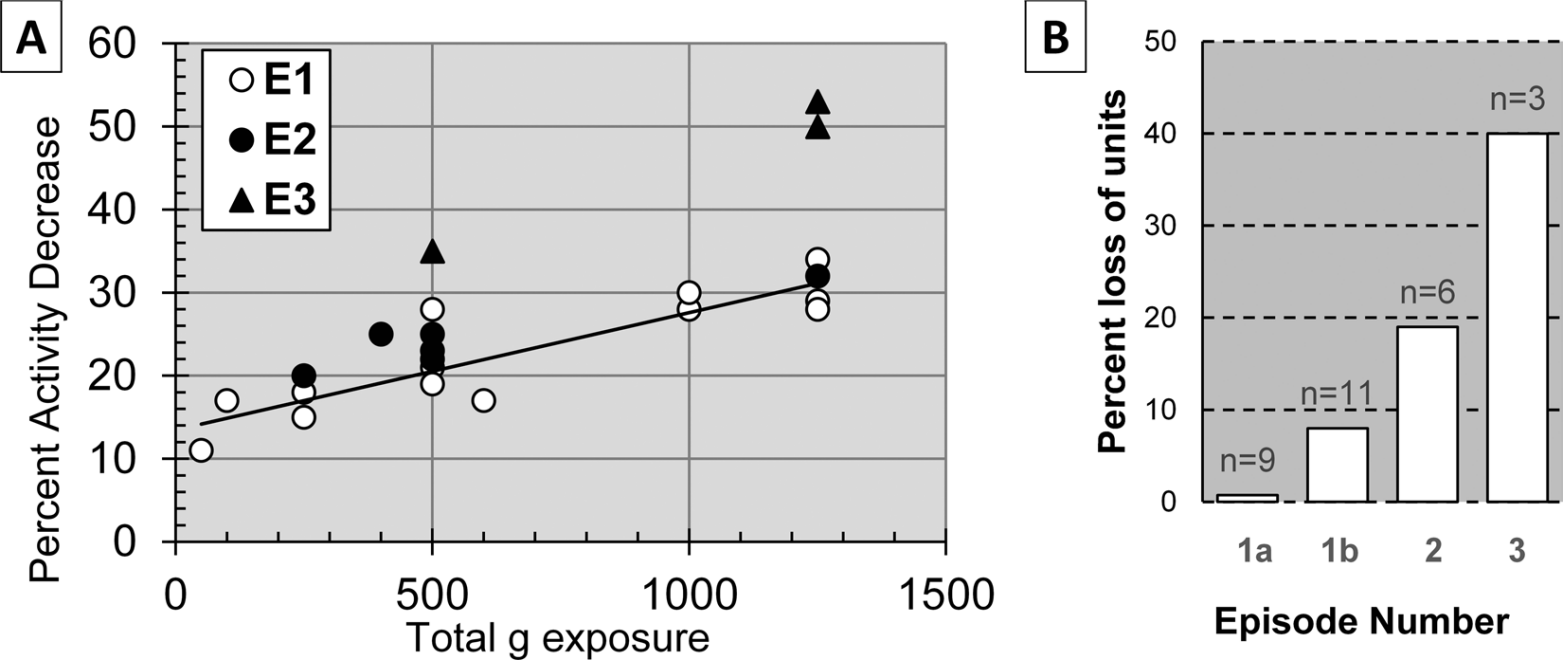
Plateau 1 deficits and percent unit loss as a function of impact episodes separated by up to 32 h. **(A)** Compounding electrophysiological deficits as a function of episode specific peak g-exposure. Episode 2 (filled circles) and episode 3 (filled triangles) data all fall above the episode 1 trend line. (**B**) Average unit loss based on episode-specific pre-impact reference data; n-values refer to the number of networks that were exposed to 1, 2 or 3 impact episodes. The first column (1a) shows episode 1 without two high unit loss experiments; the second column (1b) has ER10 and ER30 included (see Table 1).

To determine whether networks recover from their first impact episodes or show compounding effects, some of the networks of Table 1 and Fig. 2 were exposed to a second set of impacts several hours after the first episode. These results are shown in Fig. 3, which repeats data from the first impact episodes but, in addition, shows data from 2^nd^ (filled circles) and 3^rd^ (filled triangles) impact episodes as a function of episode-specific g exposure. This data display suggests that networks do not recover between impact episodes (range: 30 to 2,300 min) but show increasing damage during the 2^nd^ and 3^rd^ episodes. A full recovery between episodes would show response characteristics of the first episode exposure (open circles). This is not the case. All episode 2 and 3 data fall above the episode 1 trend line. Note that the g-values listed represent the sum of g exposures given in episode 2 or episode 3 and not the total per network. Also, the percent activity loss is based on pre-episode reference activity and not on the original native activity. These values were chosen to reveal compounding effects of multiple exposures.

The loss of electrophysiological contact (active units) shows substantial increases with second and third impact episodes (Fig. 3B). These observations can reflect three causes: a shift away from an electrode due to changes in adhesion, temporary cell inactivation, or cell death. The latter was not an overt morphological observation during the first impact episode experiments, and is confirmed by the stability of active unit counts over many hours after impact (see Fig. 1). These units are plotted every minute together with the normalized network spike activity. The degree to which cell death contributes to the data shown in Fig. 3B is not clear at present. It must be examined quantitatively with histochemical methods. However, the emphasis of this paper is on the initial g-exposure where activity decreases and recoveries occurred with stable sets of neuronal signals in 66% of the networks (no loss of units, Table 1). 83% of the networks showed less than 5% loss of units.

### Changes in network dynamics revealed by cross-correlations

In addition to the activity changes shown in Figs 1–3, there are obvious as well as subtle changes in the network firing patterns associated with exposure to rapid acceleration. As a first step, we have selected cross-correlation analysis to show that there are changes between reference and post-impact data in oscillation frequencies and firing patterns. However, the reference cell in a network environment is a statistical marker that reveals how other cells in the ensemble fire relative to the reference spikes without necessarily revealing direct interactions.

**Figure 4:**
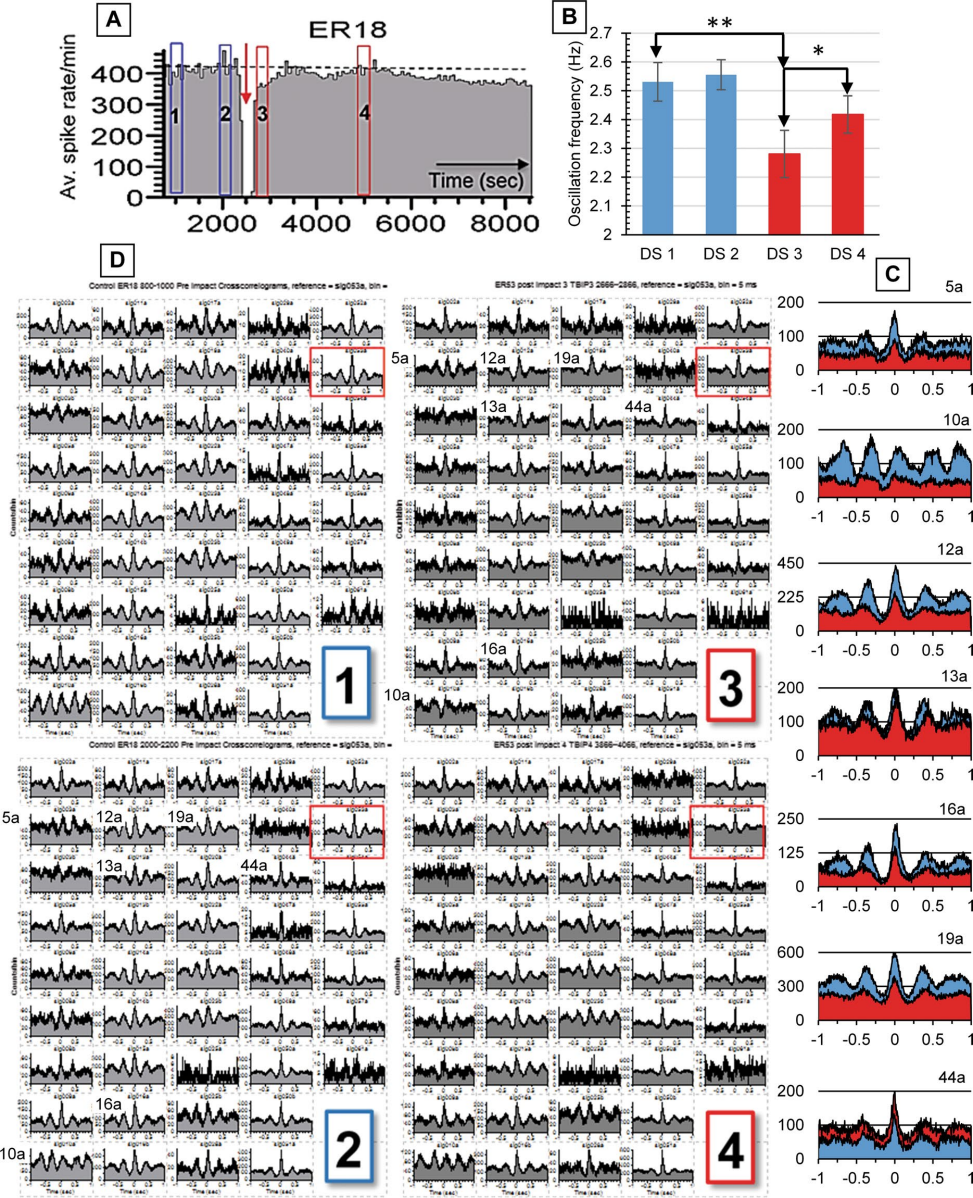
Cross correlations of separate 200 second data segments from 43 channels of network activity before (1 & 2) and after (3 & 4) impact. **(A)** Population vector of all units (average spike activity per min). Data segments 1–4 are separated by 20, 10, and 35 min, respectively. (**B**) Oscillation frequency changes; subset of 23 units; **indicates p < 0.001. **(C)** Enlarged profiles from segments 2 (blue) and 3 (red) showing amplitude and frequency changes. **(D)** Display of all 43 individual units. Panels 1–4 correspond to the respective data segments in (**A**). Reference cell is identified by the red rectangle in D.

Figure 4 shows results from 4 data segments (DS), each 200 sec in duration, before and after a single 250 g impact using all 43 recorded units. An 11% reduction in network oscillation occurs in plateau 1 (DS 3, approximately 6 min after impact), followed by a partial recovery after 35 min (DS 4). This network (ER18) had no loss of selected units and was subjected to one of the lowest accelerations of this study. The network formed plateau 1 for 6 min at 15% below reference, and plateau 2 near reference (−5%; Table 1). The population vector from all 43 discriminated units is shown in Fig. 4A together with an outline of the data segments. Panels 1–4 in (D) show all units from corresponding time segments with bin sizes of 5 ms in a ± 1 sec window. In this time range, networks reveal oscillatory activity where peaks reflect coordinated bursting or periodic increases in spike density. Panels 1 and 2 are quite similar and represent stable reference activity. However, changes in peak amplitudes and in oscillation frequencies can be seen in panel 3. Decreases in peak amplitude reflect a reduction of spikes in clusters and more random spiking between such clusters. A one-way analysis of variance and subsequent Scheffe post hoc analysis revealed significant decreases in oscillation frequencies (Fig. 4B). Post impact oscillations were reduced from an average ± SD of 2.53 ± 0.07 Hz to 2.28 ± 0.08 Hz (n = 20; p < 0.001). A partial recovery of the oscillation frequency is seen in DS4 (mean: 2.41; n = 20; p < 0.001). Selected profiles showing amplitude changes and peak shifts between data segments 2 to 3 are depicted in panel C. In many cases the second oscillation peak is missing after impact (DS 3), which represents plateau 1, but recovers partially in DS 4, representing plateau 2.

Oscillation changes occurred in all networks that were exposed to tangential acceleration. A summary of oscillation frequencies obtained from visual measurements of peak positions in cross correlations is presented in Table 3, which lists oscillation changes in six experiments and three controls. Three of the experiments analyzed to date were exposed to 40 uM bicuculline to demonstrate that oscillations are present even when GABA receptors are blocked (see discussion). It is evident that frequencies decrease after impact. Such changes are not seen in control experiments that mirror all disconnect manipulations (from amplifiers and medium flow circuit) with the exception of an impact. However, both the native oscillation and the percent decrease after impact differ for networks in medium with and without bicuculline. Networks disinhibited with 40 uM bicuculline revealed slower reference oscillations (0.5–0.61 Hz), representative of the strong coordinated burst pattern. After impact, oscillations decreased to the range of 0.4–0.46 Hz.

**Table 3:** Summary of Network Oscillation Measurements.

EXP #	Peak g/imp	# of imp	Total g	Units Lost	Hz Before ± SD	Hz After ± SD	% change + SD	n	B uM
ER05	250	1	250	0 (0%)	1.50 ± 0.03	1.27 ± 0.07	−16% ± 6	5	0
ER10	250	5	1250	18 (30%)	1.08 ± 0.02	0.88 ± 0.01	−20% ± 3	5	0
ER18	250	1	250	0 (0%)	2.53 ± 0.07	2.26 ± 0.08	−11% ± 3	20	0
ER24	200	5	1000	0 (0%)	0.50 ± 0.01	0.40 ± 0.01	−20% ± 1	5	40
ER50	250	5	1250	0 (0%)	0.61 ± 0.03	0.46 ± 0.04	−25% ± 6	5	40
ER52	250	5	1250	1 (2%)	0.58 ± 0.03	0.45 ± 0.01	−23% ± 2	10	40
**Control**									
ER55A	0	0	0	0 (0%)	0.85 ± 0.02	0.86 ± 0.02	+ 1% ± 2	5	0
ER56A	0	0	0	0 (0%)	0.66 ± 0.01	0.66 ± 0.01	0%	5	40
ER56B	0	0	0	0 (0%)	0.63 ± 0.01	0.64 ± 0.01	+ 1% ± 1	5	40

Imp: impacts;units: template-matched neural signals; **B** bicuculline, added to medium after flow system assembly. A, B In Control under EXP # designate separate networks on a 2-matrix MEA (CNNS M-5) in the same chamber; n: number of neurons sampled.

**Figure 5:**
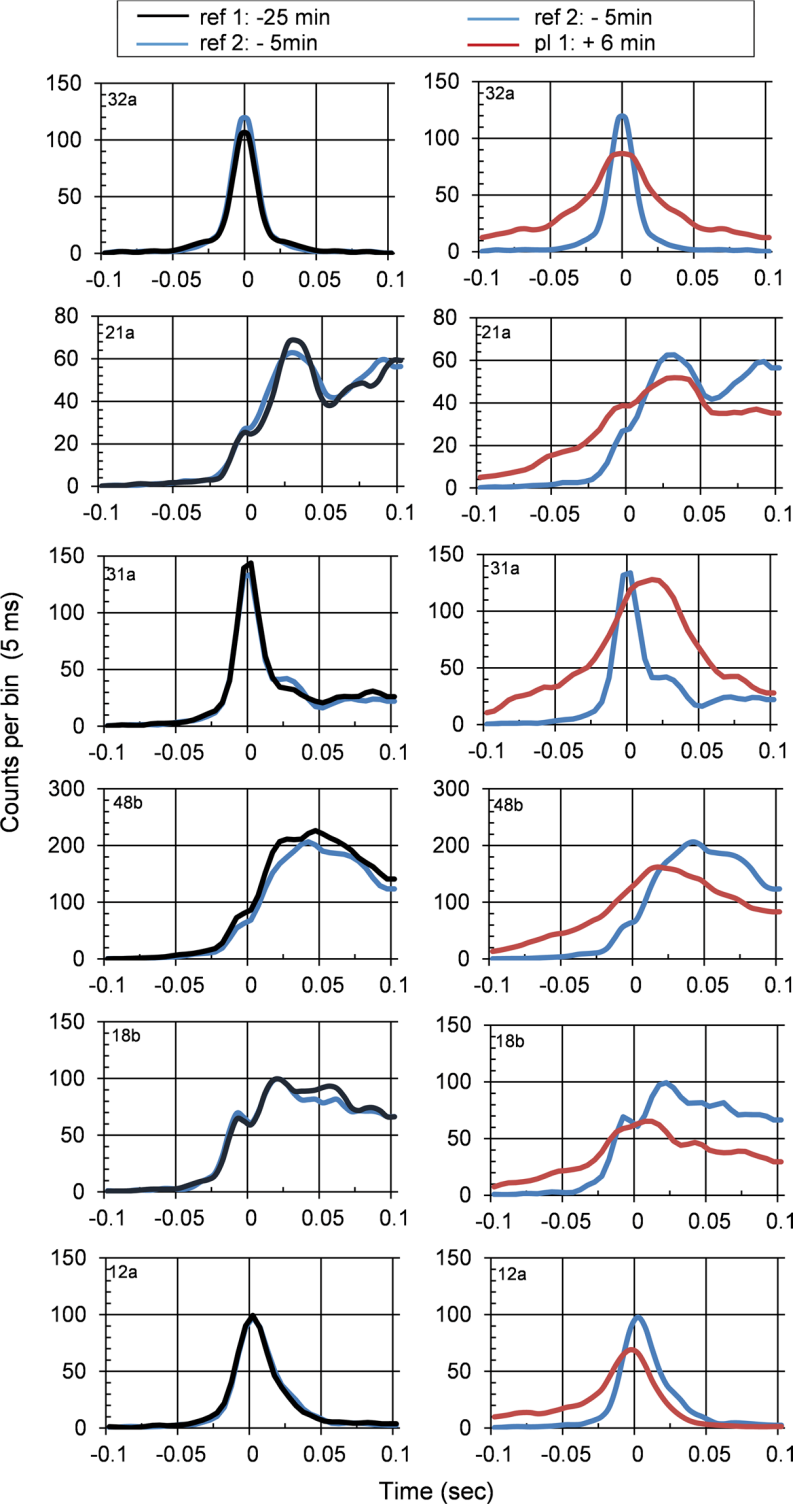
Cross-correlation profiles (CCPs) for a short time windows of ±100 ms, derived from 200 sec time segments at 25 and 5 min before impact (left column) and at −5 and +6 min after impact (right column). The reference cell (sig 32a) is shown in the first row. CCP profiles in the reference state (at −25 and −5 min) are almost identical, but spike distributions change substantially after impact. Data: ER 52 (Fig. 3).

In addition to changes in oscillation frequencies, alterations in firing hierarchies were also observed. Figure 5 shows five samples plus the reference cell from network ER 52 using three 200 sec recording segments: two from the reference period at −25 and −5 min before impact (0 min) and a time segment 6 min after the impact (plateau 1). Minimal profile changes are seen during the reference period (left column in Fig. 5), but major changes occur after the impact (right column). The −5 min reference profiles are repeated in this column to allow a direct before and after comparison. The shifting of profile peaks relative to the 0 point reflect changes in peak spike densities relative to the reference cell. A profile broadening indicates reduced organization through increased spiking between bursts. The reference cell (first row) reveals this reduction in burst organization. Unit 31a (row 3) shows a 20 ms shift of maximum spike densities.

### Morphological responses

The 30 morphological experiments performed included four reference experiments (300 to 1,000 min) and 7 incomplete experiments due to MEA breakage on impact. Of the remaining 19 complete experiments, only one showed a stable nucleus after impact; the other 18 showed unequivocal nuclear movement. In this data set, six are well-documented with time lapse data using 10 min intervals. The remaining 12 consist of still images that did not follow a strict time protocol. In 14 experiments, the nuclear motion was counter- clockwise. With the impact applied always from the right side, this motion can be considered ‘away’ from the force. Only one experiment yielded a relatively small clockwise motion (15 deg), and two experiments revealed oscillatory responses. In addition, two further experiments showed rotation out of the focal plane and could not be easily quantified.

**Figure 6:**
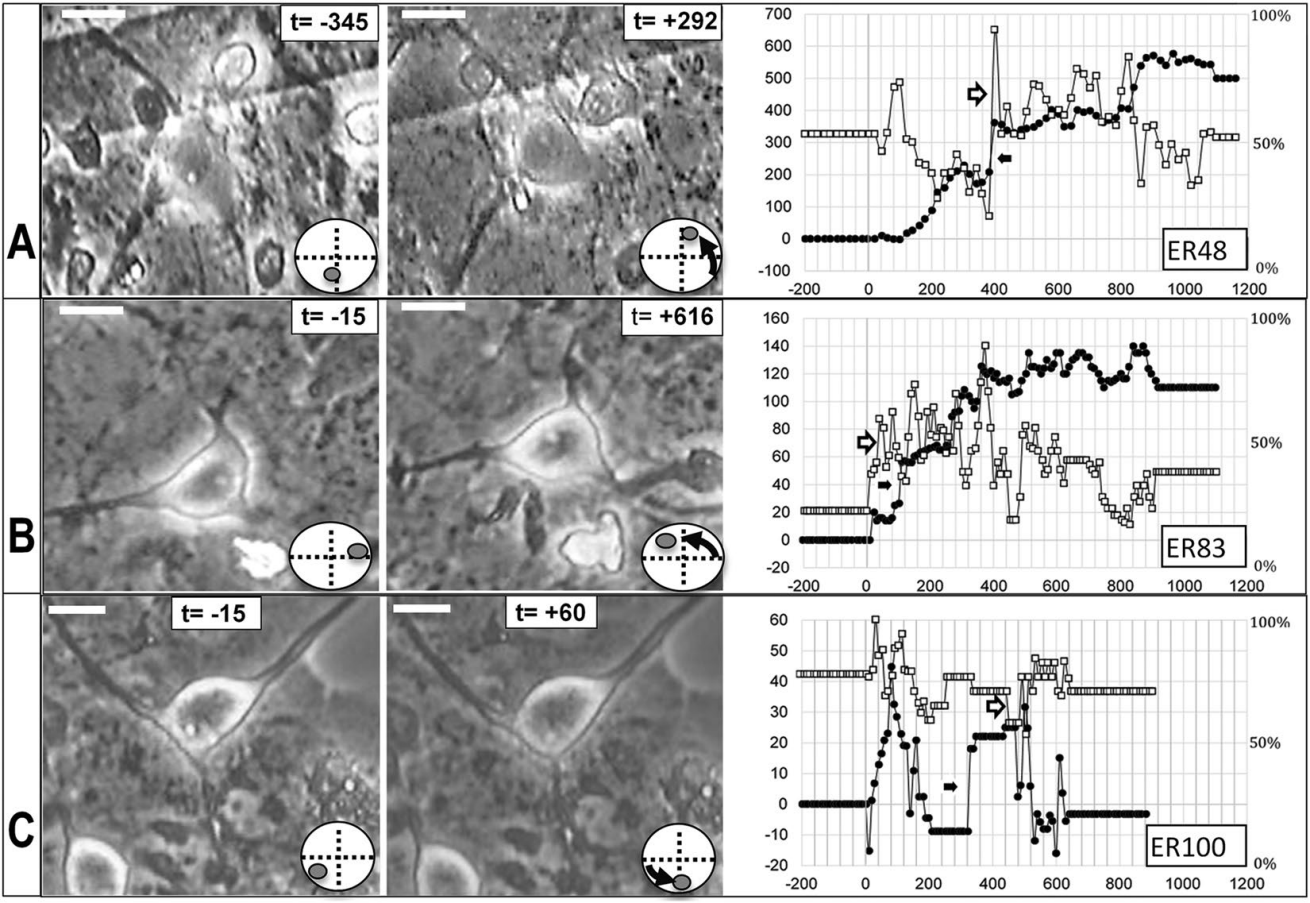
Nuclear movement in different neurons from three networks subjected to tangential acceleration. Three panels show neurons t-minutes before and after impact (t = 0) with quantitative graphs of nucleolar movements in the right column. Movement is quantified in terms of degrees of rotation in a horizontal plane (left ordinate, closed symbols) and centrifugal and centripetal drift expressed as percent of the nuclear radius (right ordinate, open symbols). Positive and increasing degree values indicate a counter clockwise displacement from origin, while negative and decreasing values represent a clockwise motion. In all cases movement starts within 30 min of impact. Transitions between fast, slow, and no rotation are occasionally accompanied by sudden jumps (arrows). Nucleolar drift is irregular whereas rotation is more consistent. Direction of impacts: right to left. Five multiple impacts were administered in 5 to 8 second intervals at 250 g each. Bar size: 20 um.

Data from three time-lapse experiments are shown in Fig. 6. The graphs attempt to quantify rotation in the horizontal plane as well as more complex precession movements using the nucleolus as the primary reference point. The measurements assume that the nucleolus is stable within the nuclear matrix, an assumption that has support in the literature^11,12^.

It is also evident from time lapse movies (see file attached), that internal nuclear features rotate with the nucleolus. Rotations are shown as positive numbers on the left ordinate, whereas nucleolar drifts toward and away from the nucleus center are shown on the right ordinate.

Observations are plotted prior to impact (at ‘0’) for 200 min to show the high stability of the nucleus in the native state. It is interesting that positive rotations (counterclockwise) dominate. With the acceleration from right to left, elastic components of the cytoskeleton may be stretched in the horizontal plane and lead to nuclear movement.

All movements start within 30 min after the impact. The rotations reveal several phases of sudden motions followed by periods of stability with small oscillations. The fastest rotations range from 3 to 9 deg per min (Fig. 6 open arrows). Other rotations out of the horizontal plane were estimated with the measurement of per cent of nuclear radius. Translated to degrees, those movements are comparable to those in the horizontal plane (2–4 deg/min, closed arrows). In addition, several observations (n = 3) of nucleolar disappearance from the optical plane reflect rotations about a horizontal axis. This presents a picture of complex rotations around many axes. The sudden transitions present in all cases imply a response to internal stresses among a variety of anchoring proteins breaking loose and attempting to re-anchor. Nuclear motions after 1200 min have not been quantified, but major changes are no longer seen. Swelling and cytoplasmic granularity are also apparent implying calcium ion entry or release from internal stores.

Initial nuclear rotation is delayed from impact by an average of 20 min (range 10 to 60 min). These observations imply that there is no immediate cytoskeletal damage, but a more gradual loss of connection between the nuclear envelope and the cytoskeleton. This surprising subcellular phenomenon is shown in Fig. 6 where the time of impact is “0” with negative time indicating observations prior to impact. Neurons and glia show morphological stability even at high g exposures during the first episode using visual evaluations over periods of 10 to 24 hrs.

## Discussion

It was our intention to develop a platform allowing simultaneous measures of electrophysiological and morphological changes in nerve cell networks for hours to days after impact injury with quantitative comparisons to hours of reference data on the same network. Extracellular recording from many sites in the network was accomplished by using MEAs with 64 microelectrodes on optically flat glass plates featuring transparent indium-tin oxide thin film conductors to provide maximum optical access. The seeding of embryonic cortical tissue from mice onto surfaces decorated with polylysine and laminin generates strong cell-surface adhesion that withstands tangential accelerations of several hundred g’s at the extremely short impact times of approximately 200 us. The cell pool consisted of all neuronal and glial cell types found in the parent tissue, and the formation of a glial carpet, consisting mostly of endothelial cells and astrocytes^13^ seems to be primarily responsible for the strong adhesion. The ballistic pendulum tangential impulse (impact) creates relatively clean biomechanical acceleration forces that stress cellular components and subcellular mechanisms.

The rapid acceleration of a fluid-filled compartment with stainless steel walls and no air bubbles is not expected to develop hydrodynamic shear stress. Only the 18 mm diameter optical window, consisting of standard 70 um thick cover glass, may undergo some deformation. Breakage of this window has not occurred under any g exposure, implying minimal movement. Under these assumed stability conditions, the elastic tissue is subjected to relatively pure acceleration shear stress parallel to the adhesion surface. The associated strain that deforms the tissue is difficult to assess. Shear shockwaves may occur, but will be difficult to measure^14^. This phenomenon has recently been described for porcine brains embedded in gelatin^15^. Of interest is their observation that the shear shock wave intensifies and peaks at an amplitude over 9 times the original acceleration (449 m/s^2^ to 4,200 m/s^2^; 45.8 to 428 g).

The injury profiles shown suggest that the substantially reduced spike activity despite minimal or - in most cases- no loss of active channels, reflect network damage. This can be categorized as “functional damage”. The functional damage has both immediate and delayed signatures (see Fig. 1) that were identified as Phase 1 and Phase 2. Whereas Phase 1 probably reflects rapid (but partially reversible) cellular and/or synaptic damage, Phase 2 shows a delayed gradual activity decay after an initial almost full recovery, implying separate mechanisms.

The lack of quantitative cell death data is a weakness of this paper. However, the influence of cell death on network dynamics *in vitro* (and *in vivo*) is complicated by the phenomenon of fault tolerance, which is poorly understood. It appears that many units (individual neurons) can be lost before basic pattern generation is changed or the network enters catastrophic failure. Pattern complexity is much more difficult to quantify and could not be accomplished at this early stage of the investigation. Consequently, as a first step, we relied on reporting the loss of electrophysiological signatures after impact (Table 1). This was minimal. In 8 of 12 experiments (66%) all original AP signatures were recovered when amplifiers were switched on after the first impact. Two of the remaining four experiments showed the loss of only a single unit. High losses of 34% and 28% were seen in only two experiments that received maximum accelerations. Yet there were deficits in AP production and network oscillations that produced the characteristic profiles shown in Figs 1, 2 and 4 and related Tables. If these activity changes were caused directly by the death of hidden units, then why does the network AP production recover to near reference in 5 to 14 min (Table 1) and why is there a partial recovery of network oscillation frequency (Table 3) approximately 40 min post impact (Fig. 4B)? These observations suggest that changes in spike production and spike organization occur either in the absence of hidden unit cell death or in spite of such cell death.

Other studies have seen similar patterns of TBI-related network activity deficits *in vivo*. Johnstone *et al*.^8^ positioned microelectrodes in the sensory cortex of rats after impact and measured responses to whisker movements. They demonstrated an initial depression in cortical network activity, followed by a recovery period, and culminating in a continual functional activity deficit measured at 24hrs. In addition, using the same methodology, Ding *et al*.^7^ reported similar network activity deficits 5 to 20 minutes immediately following injury application. The overlap with the phase 1 and phase 2 deficits reported in this manuscript is interesting. Functional damage with minimal cell death has also been reported for hippocampal slice cultures^16^. This paper takes the next step: to show functional damage from a large pool of spontaneously active neurons in a system that also allows simultaneous optical monitoring.

The rapid application of multiple impacts (5–8 sec between impacts) was dictated by frequent MEA breakage with the concomitant loss of the network and post-impact data. Insults from single impacts at 250 g are not expected to be identical to 2 quick impacts at 125 g each. However, the increase in activity reduction seen as a function of total g exposure (Fig. 2) is encouraging and justifies future efforts to improve the stability of the target arm/chamber unit. The multiple episode exposures, separated by hours, must be also be distinguished from single episode insults that were completed in less than 30 sec. Here we saw an enhancement in plateau 1 decreases as well as greater loss of electrophysiologically active units.

### Network oscillations

Networks *in vivo* generate several oscillation bands at frequencies ranging from 0.05 to 500 Hz^17^. Oscillatory activity appears ubiquitous in mammalian brains. It is seen at the sub- and supra-threshold levels, is activity-dependent, and is assumed to have functional significance. Using magnetoencephalographic connectivity measurements, Vakorin *et al*.^18^ observed that mTBI in human subjects at an average of 32 days after injury can be linked to ‘reduced network connectivity’ in frequency ranges above 30 Hz. It is interesting, that oscillations also appear in hippocampal slices *in vitro*^19–21^ using cholinergic^19^ and kainate induction^20,21^. We have observed a common rhythm in primary cortical cultures in a range from 1.5 to 3.0 Hz in the native state without chemical induction and at the biological temperature of 37 °C. Blocking the primary inhibitory synaptic influence with 40 uM bicuculline decreased the spontaneous frequency range to 0.5 to 1 Hz. This range falls into the delta class of brain oscillations (1.5–4 Hz)^17^ that is known to arise in the thalamus and in the cortex. Previous *in vitro* studies have implicated phasic inhibitory transmission and participation of electrical synapses^20,22,23^. The fact that oscillations are observed in disinhibited networks implies inhibitory neurons are not necessary for the genesis of delta oscillations. It has already been suggested that the precise and long-lasting burst oscillations seen in synaptically simplified spinal cord networks *in vitro*, in which only NMDA-receptor mediated excitation remained, were controlled by presynaptic vesicle release mechanisms^24^. However, our main point in this paper must be that the oscillations, regardless of origin, are present and can be used to quantify damage. Oscillation measurements could be very useful to establish time scales of recovery, required for investigations of pharmacological interventions.

### Nuclear rotation and suggestions for damage mechanisms

Whereas the primary damage can be “visualized” by invoking either microporation^25^ - a phenomenon that reduces membrane potentials and may block activity - or disruption of exocytosis mechanisms at presynaptic terminals, an explanation for the secondary damage is more difficult. However, the nuclear rotation, which has emerged as a consistent morphological response to impact, is pointing to a potential secondary damage mechanism. Neural cells are highly polarized cells with different functions residing in pre- and post-synaptic regions^26,27^. Protein export is thought to be controlled by specific trafficking mechanisms^28^. Nuclear stability plays a critical role in many cellular and developmental processes and is essential for proper cell function^29^. Nuclear rotations are likely to disturb this transport specificity resulting in protein deficiencies in target regions. Such deficiencies develop slowly, depending on the gradual loss of regular transport cargo. This interpretation agrees with the activity loss seen in the dominant 2-phase response. Although such trafficking problems in neurons have received attention^27^ they have not been linked to impact injuries. Puzzling is still the delay between impact and the beginning of nuclear movement. There is no immediate shifting of the nucleus and initiation of rotation occurs in a time frame ranging from 10 to 60 min. Whether these delays are a function of impact intensity is not clear. However, it is reasonable to speculate that Ca++ entry and/or internal release is involved in the nuclear rotation phenomenon. Calcium ions were also implicated in a report on nuclear rotations in dorsal root ganglion cells^30^.

It was realized already in 2011 that functional changes can occur “on the neuronal network level even in the absence of histologically significant injury”^7^. There is no evidence that a disruption of protein trafficking will lead to certain neuronal death, however there is emerging evidence that neuronal functions are disturbed. The development of this BPA system will provide an economical, sensitive, quantitative test platform to achieve such correlated observations. Although the present system loses about 4 min of data immediately after the impact, a time period which is critical for a complete understanding of rapid acceleration damage, it is technically feasible to combine the BPA with a microscope so that high power microscopy and fluorescence microscopy are possible within 10 to 20 sec after the impact.

The repeatability of the response profiles and changes in network oscillations is encouraging and suggests that systematic investigations of chemical and physical interventions, to enhance recovery of neuronal functions, will be possible. Classification of subtle damage and functional deficits into immediate, early, and delayed stage responses would provide a link to *in vivo* data. *In vivo* biochemical changes, such as the generation of acrolein^31^ and microglial activation^3^ could also be duplicated *in vitro*. Additionally, this method could provide a unique bridge between the study of how cells sense and translate mechanical forces and deformations in their physical environment and investigations of associated biochemical signals and genetic alterations^5,32,33^.

## Methods

**Figure 7:**
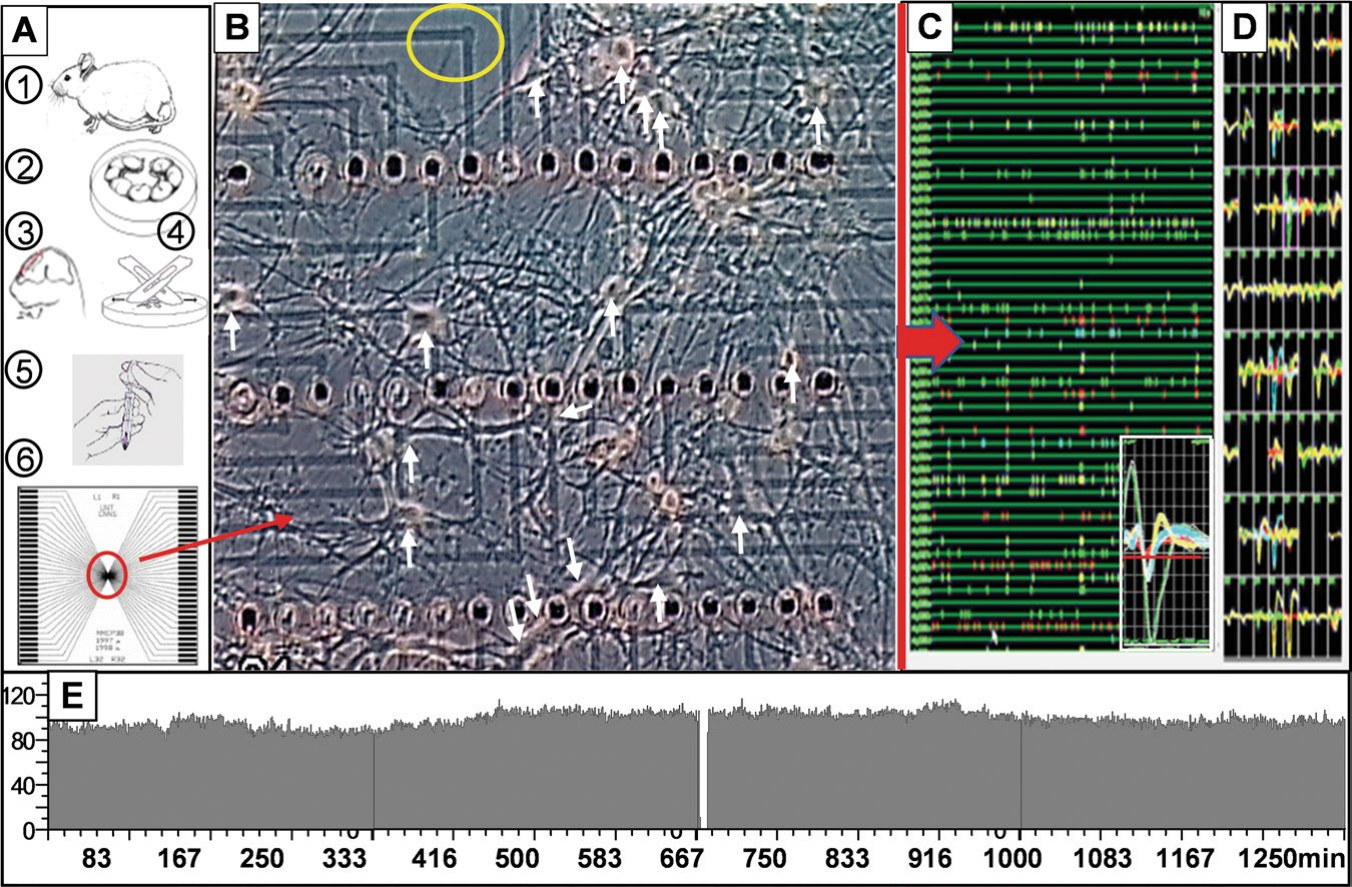
Summary of major steps involved in cell culture and recording. (**A**) Tissue preparation and seeding onto MEA. A1: Timed pregnant mice were obtained on gestational day 16 on arrival and processed on day 17. A2: The embryonic sack was removed under terminal anesthesia and washed several times in iced calcium-free solutions (D1SGH). A3&4: Cortical tissue was removed from each embryonic brain, pooled, and minced with scalpels. A5&6: After trypsin digestion, the tissue was triturated, centrifuged, and adjusted for cell concentration with culture medium before seeding. (**B**) Phase contrast micrograph of a live network at 22 days *in vitro* (d.i.v.) on a recording matrix. Vertical electrode spacing: 200um, lateral spacing: 40um. Only three of four electrode rows are shown. Numerous large neurons (>20 um) diameter are identified by arrows. The transparent indium-tin oxide thin film conductors are 10 um wide. (**C**) A 30 sec segment of the continuous raster plot displayed by the Plexon Rasputin program after amplification and digitizing. Action potentials templates allow separation of up to 4 signals on one channel in real time (insert). (**D**) 64-channel matrix with selected templates displayed continually. Different colors identify selected templates on specific channels. (**E**) 22 hrs of control data. NEX display of average network activity/min for 11 hrs before and after a sham impact (gap at 667 min shows disconnect from amplifiers). Drawings in (**A**) by Emese Dian^58^ with permission. License link: (https://creativecommons.org/licenses/by/4.0/). Components of drawing were rearranged from original^58^.

A pictorial summary of the major cell culture and recording steps required to obtain electrophysiological data from networks is shown in Fig. 7. Neural tissue from gestational day 16–17 mouse embryos were used to generate spontaneously active networks that are considered mature at 3–4 weeks, based on stable activity patterns and reliable pharmacological responses^34–37^. We used embryonic tissue to ensure that the resulting networks would contain a mixture of neuronal and glial cell types representative of the parent tissue. Extensive pharmacological data gathered over the past two decades by numerous laboratories have shown such protocols to generate histiotypic network activity that mimics responses of the parent tissue^38–40^. For the research presented, all procedures were approved by the University of North Texas Animal Care and Use Committee.

The recording matrix in Fig. 7B shows three of four rows of electrodes and depicts a low-density network region at 23 days *in vitro* (d.i.v.) with approximately 20 large neurons and numerous smaller (<20 um) neurons. The seeding of cells produces a random distribution of cell types with a glial cell layer generally below the neuronal cell bodies. Such unconstrained networks seem to have a higher fidelity of information transmission than ordered networks with several nodes^41^. The carpet is not complete and shows an open area above electrode row 1 (circle). Signals are obtained from axons, cell bodies, and possibly dendrites^40^. Axonal signals dominate, and such processes have been found above and below the glial layer. If trapped by glia in the shallow recording craters, large signals approaching 1 mV in peak-to-peak amplitudes can be obtained. Control activity is shown in Fig. 7E from 4 consecutive 20,000 sec NEX data files (Neuroexplorer, Nex Technologies). Small fluctuations occur but average activity is maintained even after 22 hrs in the recording chamber.

### Preparation of microelectrode arrays

Microelectrode arrays were fabricated in-house using the methods described^42,43^. MEA conductor patterns consisted of 64 transparent indium tin oxide electrodes arranged as a single or dual recording matrix configuration^44^. The latter design allows cultivation of two age and maintenance-matched but separate networks, each growing on a 32-electrode recording matrix. Conductors were laser de-insulated at their terminals creating shallow recording craters, approximately 1.5 to 2 micrometers deep, that were electrolytically gold-plated to lower interface impedances to approximately 0.8 megohms. The methyl-trimethoxysilane resin insulation was flamed^45^ through masks for localized surface activation and subsequently coated with poly-D- lysine and laminin.

### Cell culture

Frontal cortex tissues were dissociated from E-16 embryos of ICR mice (Envigo, Inc.) following the pioneering protocols of Ransom *et al*.^46^ with minor modifications^24^. Cortices were minced mechanically, enzymatically digested with trypsin, triturated, combined with Dulbecco’s Modified Minimal Essential Medium (DMEM), supplemented with 5% fetal bovine serum (SAFC Biosciences) and 5% horse serum (Atlanta Biological), and seeded at 50–70k cells per 100uL onto MEAs (~3 mm diameter adhesion island; ~7 mm^2^ area), yielding approximately 300 neurons per mm^2^ (~2,100 total) on a carpet of glial cells after network formation (see Fig. 7). The original cell pool consisted of all cells found in the parent tissue (neurons, astrocytes, oligodendrocytes, microglia, and endothelial cells from disrupted capillaries.

Cultures were maintained at 37 °C in a 10% C0_2_ atmosphere and, after two days, were transitioned into medium containing 5% horse serum. Medium changes were performed biweekly. Under optimal conditions the resulting neuronal networks can remain spontaneously active and pharmacologically responsive for many months^47–49^. If an average of 60,000 cells are seeded and if it is assumed that 1/3 are differentiated neurons, we can expect 20,000 neurons to be dispersed in a 7 mm^2^ area (~2,800 neurons per mm^2^). Substantial attrition occurs in the first two weeks, with stabilization at approximately 3 weeks based a subsequent neuronal loss of approximately 3% per month^18,39^ and on repeatable pharmacological/toxicological responses^24,39,40,50,51^.

### Chamber and life support

**Figure 8:**
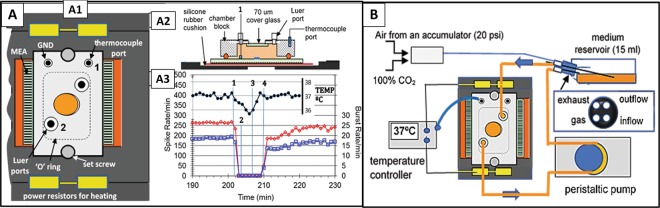
Recording chamber and life support system. **(A)** Assembled recording chamber with temperature measurements during experimental protocols. (A1) Base plate, MEA, and assembled chamber block. (A2) Side view of chamber with thermocouple placement for medium and chamber temperature measurements. Medium temperatures were measured through a Luer port at position 1 in separate tests. Chamber volume: 5 ml. (A3) Medium temperature before, during, and after system disconnect with correlated mean spike and burst rates. (1) Recording and life support disconnect; (2) impact; (3) life support and amplifier reconnect (4). Amplifier power on. Note that the recording period was started only when the temperature had reached reference. **(B)** Life support system. A peristaltic pump moved medium at a rate of 0.5 ml/min from a heated reservoir containing 15 ml of medium through the chamber and back into the reservoir. 10% CO_2_ (10 ml/min) was provided by an Aalborg gas controller from mixing 100% CO_2_ with filtered room air compressed in an accumulator at 20 psi. A DC temperature control system maintained the chamber temperature at 37 ± 1 °C via four 4-ohm power resistors. Total system medium volume: 20 ml.

The assembled chamber holds the MEA and allows life support functions to be maintained while connected to the amplifiers on the microscope stage. The recording chamber consisted of a base plate which holds and heats the MEA via four power resistors (37 °C ± 1°) and a chamber block which contains a microscope window as well as medium access conduits Fig. 8. Medium enters and exits via Luer access ports. The chamber has a maximum volume of 5 ml to minimize pH and osmolarity fluctuations and reduce network disturbance from supply line manipulation. Preamplifiers (Plexon Inc., Dallas, TX) were connected to each side of the chamber via edge connectors and zebra strips^47^ (Fujipoly America Corp, Cartaret, NJ).

Network activity is temperature sensitive and a stable medium temperature within ±0.5 °C of a set point (37 °C) is desired. During recording, a thermocouple was always attached to the metal chamber block to monitor temperature in real time. Medium temperature was measured in special tests. A preheated BPA (with a heat lamp) maintained medium temperature within 1–2 °C of reference, which recovered on the microscope stage within 2–3 min (Fig. 8A). Consequently, a delay time of 3 min before amplifier activation was adequate for system recovery to the set point temperature. This protocol ensured that activity measurements did not reflect temperature effects but represented impact-related changes in network dynamics.

A closed life support system was used for pH control and nutrient supply for the culture (Fig. 8B). This system consisted of a culture flask containing 15 ml of culture medium, supply lines, a peristaltic pump (set to 0.5 ml per minute), and connectors to the chamber block. Rapid and convenient sterile connection with the supply flask was obtained via an autoclavable needle assembly (insert in B). The rubber stopper holds a total of four 21 ga needles for medium pick up, medium return, gas supply (10% CO_2_ in air), and exhaust. Sterile assembly of all tubing and system priming is almost impossible without this device. The reservoir was positioned 20 cm above the chamber to compensate for under-pressure created by the peristaltic pump and was heated to 40 °C to enhance outgassing and minimize bubble formation in the chamber^52^. Water injection directly into the Pharmed rubber tubing return line via a syringe pump (~50 µl/hr) maintained osmolarity in the reservoir.

### Impulse (Impact) generation

**Figure 9:**
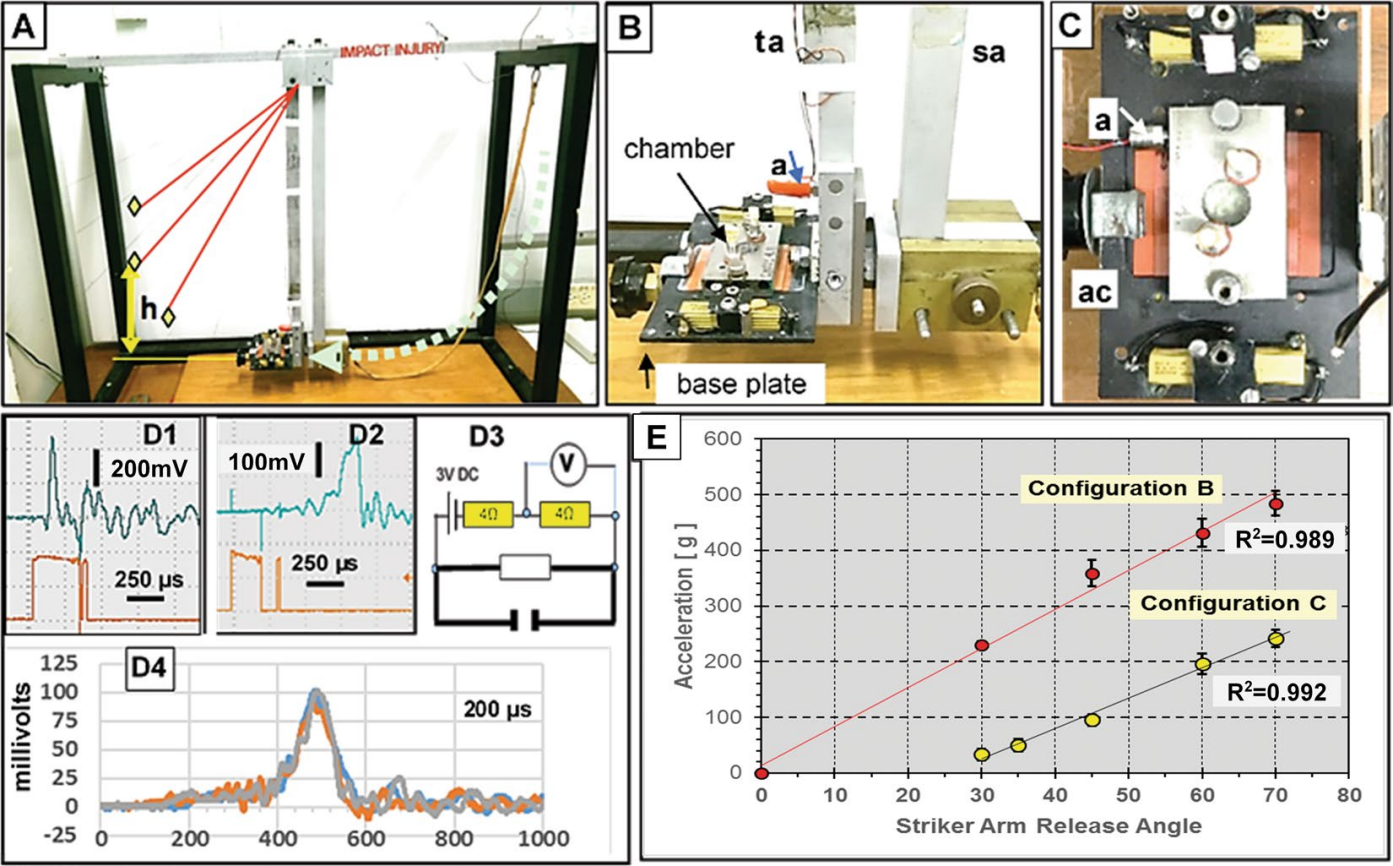
Impact generation and measurement. **(A)** Ballistic pendulum apparatus (BPA)- The striker arm (sa) swings down from a preselected height (curved arrow) making contact (impact) with the target arm (ta) which reaches a height of “h” (vertical arrow). H reflects the kinetic energy transferred to the target. **(B)** Base plate and recording chamber on the **ta** platform. Note accelerometer **(a)** attached to the target arm. **(C)** Base plate and chamber on **ta** platform with accelerometer **(a)** attached to the side of the chamber in line with the direction of force. This represents the preferred accelerometer positioning but interfered with amplifier attachment. Power resistors, plugs, and attachment clamp **(ac)** are visible. **(D** 1&2) Oscilloscope traces for simultaneous g meter (top) and electrical contact measurements (bottom trace) resulting from an initial striker arm angle of 60° for accelerometer configurations **B** and **C**, respectively. The g-meter reading is delayed 50 us by the Isotron circuit and further by mechanical yield in configuration ‘C’. **(D3)** Circuit used to record contact time (see text). **(D4)** Three separate accelerometer traces taken consecutively at 45° (Config. C) showing good profile overlap. Configuration C broadens the trace by a factor of 2 and reduces peak g’s by ~50%. **(E)** Calibration of peak acceleration as a function of striker arm angle (30°, 45°, 60° and 70°, n = 6 each) for the two accelerometer attachment configurations. Config. C yields linear readouts only between 30 and 70 degrees and reports g-levels similar to those experienced by the network. Calibration: 1.006 mV/g.

The ballistic pendulum apparatus (BPA) was selected for its ability to apply well defined tangential acceleration to networks. It consists of a striker arm and a target arm holding the MEA/chamber assembly (Fig. 9). The striker arm collides with the target arm which is accelerated to reach maximum velocity (V_max_) within a time (t) and swings up to a height (h). This movement dissipates the acceleration and minimizes secondary influences. The target arm was arrested and stabilized by hand at maximum h.

Acceleration was measured with an Endevco Isotron accelerometer, Model 7255A-1 (calibration 1.006 mV/g) monitored with a Tektronix TBS-1022 oscilloscope. Contact times were assessed by a DC circuit as shown in Fig. 9D. A 3 V DC connection was made on each arm. Because of the metal construction (aluminum), a current was constantly flowing, even when arms were separated. However, the momentarily decreased resistance during contact provided a current pulse that could be easily recorded. The g meter reading was offset by ~50 us from the beginning of the current pulse by a circuit delay in the Isotron line conditioner. The high sweep speeds made AC shielding unnecessary. The current pulse usually lasted longer than the g meter reading, implying metal/metal contact for up to 300 us after the maximum momentum transfer.

Physical design constraints associated with preamplifier and life support attachments did not allow a permanent accelerometer attachment on the chamber. Experimental g measurements had to be made with the accelerometer on the target arm (Fig. 9B). Both positions (labeled “a” in panels B and C) were calibrated with 6 impacts at each of 5 angles. The difference in readout was surprisingly large (Fig. 9E). Whereas the target arm responds directly to the impact, the chamber experiences a slower acceleration because forces are transferred from the target arm to the base plate and then to the chamber via two stainless steel set screws. The accelerometer registers further delays (range: 300–500 us) that represent the time between target arm impact and chamber acceleration. The MEA with the network is held in position by friction from a 2 mm thick silicone rubber O-ring embedded in the bottom surface of the chamber and by a thin silicone rubber mat between the glass MEA and the base plate. The friction is strong enough to prevent slippage of the MEA. Breakage occurs before slippage. The chamber acceleration is therefore representative of what is experienced by the network. Parallax errors of angle measurement were minimized by a thin rod attached to the striker (s) near the center of mass. All data are reported in terms of chamber acceleration.

The apparatus used for these experiments caused excessive breakage of MEAs above 300 g (striker arm above 70°). Consequently, with some exceptions, multiple single impacts in rapid succession between 30 and 250 g preserved MEA integrity and resulted in measurable network responses. This group of high frequency impacts was termed an “impact episode”. We selected 1–5 successive impacts separated by 5 to 8 sec at a preselected initial angle.

### Recording

Analog electrical signals were amplified with a total gain of 10 K, digitized at 40 kHz, and transformed into time stamps for storage and later analysis (Plexon Inc., Multichannel Acquisition Processor System, Dallas TX). Active units were discriminated by waveshape templates (Plexon Inc.), which allows real-time sorting and the identification of individual units. Under optimal conditions (visual signal-to-noise ratios >3:1), four different waveshapes can be distinguished on a single electrode in real time. When templates crossed an assigned threshold, spikes were logged with a resolution of 25 us. Network activity (spike and burst production) were quantified as described previously^18,24,53^. Network spike production was plotted as mean spikes per minute for the entire experiment. Each minute, the total activity was divided by the active channels (floating average). An active channel was defined conservatively as one with at least 10 discriminated spike signals per minute. Such a display allowed the monitoring of the evolution of activity and represented the primary real time contact with the network. Burst activity was monitored with the Plexon raster display and quantified offline. All recordings were conducted in normal cell culture medium containing 5% horse serum and sodium bicarbonate buffer.

Activity from different networks was never pooled, and changes were normalized as percent decreases from network-specific reference activity that was maintained in a stable state for a minimum of 1 hr (native activity) before impact experiments. Native activity in two experiments exceeded 10 hrs. Neurophysiological parameters were quantified from spike rate plots using NeuroExplorer analysis programs (NEX Technologies, Colorado Springs).

### Burst identification

Once spikes had been discriminated using wave shape templates (Plexon Inc.), the time stamps were integrated with an integration constant of 70 ms. Two thresholds were used to identify bursts: T1 (at a level close to the noise) and T2 (at 5x the threshold). T2 is set to determine whether a T1 signal was indeed a burst. A single low threshold often includes noise, and a single high threshold delays burst onset times^53,54^. Because burst termination is biased by the decay constant, a 10 ms adjustment was made to “snap” the profile closer to the last spikes of the burst. If activity remained below T1 for more than 100 ms, two bursts were generated. This gap time was adjustable, and selection depended on the overall spike pattern provided by the display of time stamps in NeuroExplorer.

### Cross-correllations (CC)

Signal analyses often use cross-correlation functions to determine differences in two waveforms by applying a time-shift to only one of them. Cross-correlations have been adapted in neuroscience to show the relationships between two action potential series from two neurons in terms of time stamps^55–57^. In isolated systems it is possible to determine direct effects of one cell on another and therewith suggest inhibitory or excitatory connectivity. Although networks in culture are isolated systems, their constant spontaneous activity makes such detailed cellular interactions difficult to determine. However, as a statistical comparison of activity states “before” and “after” a stimulus to show that changes have occurred, these approaches are useful. The algorithms generate histograms where the X-axis represents time and the Y-axis the number of response spikes that occur in preselected time bins. These calculations are performed for every reference cell spike in a selected time period. We used the NeuroExplorer V2.2 for generating the cross-correlation profiles (CCPs). On a wide time-scale of −1 to +1 sec or greater, the CCPs reveal oscillations related to bursts or periodic increases in spike densities; on a shorter time-scale, the profiles reflect a statistical view how cells fire relative to the selected reference cell.

### Morphological measurements

The transparent ITO conductors and optically flat glass plates of the MEAs used allowed real time, continual high-resolution microscope monitoring in parallel with multichannel electrophysiological recording. Phase contrast microscopy (Zeiss Axiovert 100) coupled with time lapse photography (Flashbus V.2.0) recorded still images of selected neurons for reference periods and a post-impact monitoring of up to 2000 min. Images were analyzed with Adobe Photoshop, ImageJ, and iMovie.

Nuclear rotation was measured with two parameters of nucleolar movement: horizontal plane rotation in degrees per minute and centrifugal/centripetal movement in pixels (percent of radius) relative to the nuclear center. This provided an internal reference to avoid errors due to small movements of camera, microscope stage, or cells. Positive and increasing values of nucleolar rotation indicate a counter clockwise displacement from the origin. Negative and decreasing values represent a clockwise motion.

### Control experiments

Control experiments (n = 5) followed all impact protocol procedures, including the disconnect from the recording system, removal from the microscope stage, and placement onto the BPA, with the notable exception of the application of force (no impact). Control experiments (Fig. 1E) showed minimal changes in activity (±10%), with no loss of units for approximately 200 min after reconnect. None of the control experiments resulted in activity profiles similar to those associated with impacts. Additional “non disconnect” control experiments were performed (n = 3) to demonstrate long term network stability in the chamber for days on the microscope stage.

### Statistics

This study was based on direct observations of neuronal network responses to rapid acceleration. Average values were used with standard deviations to show the variation in samples. All network activity data was plotted in one-minute bins where the total spike activity was divided by the number of neurons active in that minute. To be counted as an ‘active unit’ a template had to log a minimum of 10 threshold crossings per minute. These data points (Fig. 1) are population responses, free of variations between channels. All activity decreases (or increases) were normalized relative to the reference activity of the network under investigation and expressed as percent of reference. Oscillations of individual units (Fig. 4) were obtained from cross-correlation profiles using 5 ms bins and ±1 sec windows. Oscillation periods were measured visually from enlarged profiles using peak -to-peak distances. The focus here was on before/after changes and not on differences in the population. Statistical significance was established with a one-way analysis of variance and subsequent Scheffe post hoc analysis.

## Supplementary information

Nuclear Rotation

ER56 Control Plexon Data Part 1 of 2

ER52 Experimental Plexon Data Part 2 of 2

ER52 Experimental Plexon Data Part 1 of 2

ER56 Control Plexon Data Part 2 of 2

## Data Availability

Data files are available for all experiments upon request. In addition, a set of control and experimental representative electrophysiological and morphological raw data files have been included in the Supplementary Data Section. Network activity recordings for experimental (ER52, Fig. 1) and control (ER56) studies have been provided as a series of two ~350 minute (~700 min total).ZIP files converted from.PLX files (Plexon), which were originally analyzed using NeuroExplorer as previously described. The plots presented in this manuscript (ex. Fig. 1) are taken from VernAC, a proprietary software developed in-house to analyze network activity, but can be recreated using the data provided and most standard graphing software (i.e. Matlab, Excel, etc.). Morphological analyses were performed as previously described utilizing phase contrast microscopy combined with time lapse photography and stored as.JPEG image files. A collection of such images for ER48 is provided in the Supplemental Data Section in the form of a short video.
